# Characteristics of chronic subdural haematomas related to DOACs vs warfarin

**DOI:** 10.1186/s12883-025-04134-3

**Published:** 2025-04-26

**Authors:** Gokul Raj Krishna, Aleksandra Sobieraj, Sayan Biswas, Anand Pandit, Kate Sheridan, Anas Patel, Richa Job, Alishba Rizvi, Hannah Cheng, Maya Holt, Lameesa Kaysor, Aaliya Ashik, Joshua MacArthur, Ella Snowdon, Ved Sarkar, Callum Tetlow, K. Joshi George

**Affiliations:** 1 Faculty of Biology, Medicine and Health, University of Manchester, Manchester, England M13 9PL UK; 2 High-Dimensional Neurology, Institute of Neurology, University College London, London, UK; 3 College of Letters and Sciences, University of California, Berkeley, CA 94720 USA; 4 Division of Data Science, The Northern Care Alliance NHS Group, Manchester, England M6 8HD UK; 5 Department of Neurosurgery, Manchester Centre for Clinical Neurosciences, Salford Royal Hospital, Manchester, England M6 8HD UK

**Keywords:** Anti-coagulation, Chronic Subdural Hematoma, DOAC, Warfarin

## Abstract

**Objectives:**

The aim of this study was to investigate the effects of anticoagulation with DOACs and warfarin on the characteristics of chronic subdural hematomas (CSDHs), specifically, the size of the hematomas, the presence of midline shift and the effect on consciousness levels, measured via the Glasgow Coma Scale (GCS).

**Methods:**

A multi-centre retrospective case series analysis from January 2015 to May 2020 was conducted. Patients who were anticoagulated with DOACs and warfarin were of primary interest. The CSDH characteristics that were focussed on included the size of the CSDH, midline shift and GCS. Chi-squared analysis and independent t-tests were conducted for inter-variable analysis. Relative risk was also calculated.

**Results:**

Two thousand, six hundred seventy-five patients across two tertiary neurosurgical units referred with CSDHs were included in the analysis. 1799 patients were male (67.3%), with a mean age of 78.5 years. 905 patients (33.8%) were on antithrombotic therapy, with 298 patients (11.1%) on warfarin and 203 patients (7.6%) on DOACs. There were statistically significant associations between the type of antithrombotic medication and both midline shift and size of the CSDH (*p* < 0.0001), but not GCS (*p* = 0.1956). No significant difference in relative risk (RR) for impaired GCS was found between DOACs and warfarin (1.158 vs 1.174 respectively). Relative risk analysis revealed a safer profile for DOACs, with a lower risk of developing a larger sized hematoma (RR 0.887 v 1.021) and a reduced likelihood of midline shift (RR 0.858 VS 0.938), which was supported by effect size analysis using odd’s ratios. Comparative risk analysis between DOACs and warfarin further demonstrated a higher risk of midline shift for patients on warfarin (RR 1.431), that trended towards statistical significance (*p* = 0.0511, 95% confidence interval 0.998–2.05).

**Conclusions:**

For CSDH patients, DOACs may potentially be a safer method of anticoagulation as opposed to warfarin as they appear to be linked to the development of smaller sized hematomas and reduced midline shift, although there was no significant difference in GCS between the groups. These features are known to reduce the risk of needing neurosurgical intervention for CSDH. This is important in influencing the management of an increasingly ageing, multi-morbid population on increasing amounts of anticoagulation medication.

## INTRODUCTION

Chronic subdural haematoma (CSDH) is a common neurosurgical condition where there is a collection of blood products between the dural and subarachnoid layers of the brain, commonly associated with the elderly population [1]. It has an annual incidence rate between 8.2 – 48/100,000/year, with a 53% predicted increase by 2040 in the United Kingdom [2], as the result of an increasingly ageing population on an increasing amount of anticoagulant or anti-platelet medication [3, 4]. CSDHs often present heterogeneously, with varying symptom progression over days to weeks, and can be difficult to diagnose solely based on presenting symptoms. Therefore, predictive risk factors are a particularly useful diagnostic aid. Observational studies have shown that the use of anticoagulants increase the risk of the development and recurrence of CSDHs [1, 5], however, these studies have largely focused on warfarin as the use of direct oral anticoagulants (DOACs) was less prevalent. However, the preferential usage of DOACs over warfarin has exponentially increased in the past decade, particularly for the prevention of stroke and thromboembolisms, due to their superior safety profile, reduced monitoring requirements, favourable pharmacological profile, and reduced drug interactions [6, 7]. Whilst there is evidence that vitamin K antagonists are associated with a higher risk of subdural haematomas [1, 3], there is significant paucity in the current literature regarding the impact of warfarin or DOACs on the features of CSDHs. Features that were found to be the most important predictors of neurosurgical acceptance for considering surgical evacuation were the size of the CSDH, presence of midline shift and the Glasgow Coma Scale (GCS) score [8, 9]. Thus, the purpose of this study was to investigate the impact of oral anticoagulation, specifically comparing DOACs versus warfarin, on the characteristics of CSDHs, particularly the size of the haematomas, presence of midline shift and the effect on consciousness levels (measured via GCS).

## Methods

### Data source and feature selection

This multi-centre, retrospective case series analyzed all patients presenting with CSDH between January 2015 to May 2020. The neurosurgical referral databases at the trusts were reviewed, and all CSDH referrals were anonymously exported for analysis. A total of 2675 patients presenting with CSDHs across two tertiary neurosurgical units in the UK were identified. The study’s retrospective and blinded nature alongside the anonymized data analysis pipeline rendered patient consent not necessary.

Consent to Participate declarations were not applicable in this study. The study was approved by the Northwest Research and Innovation Institutional Review board, reference number: 22HIP11. All subsequent methods were performed in accordance with the relevant local guidelines and regulations. The study further adhered to the Declaration of Helsinki.

Data on 12 variables stored in the referral database at the time of referral was collected: age (continuous), sex (binary), ischemic heart disease (binary), cardiac rhythm disorder (binary), peripheral vascular disease (binary), cardiac valvular disease (binary), Glasgow Coma Scale (GCS) score (discrete continuous), midline shift (binary), CSDH size (small, medium and large; determined from neuro-radiological reports), antithrombotic therapy status (binary), antithrombotic medication type (categorical nominal) and total number of antithrombotic medications (discrete numerical). CSDH sizes were defined using the maximal hematoma thickness: < 1 cm being considered small, 1 – 2 cm as medium and > 2 cm as large. Primary outcome of the study was the impact of DOACs and warfarin on 3 main outcome variables: GCS at presentation, CSDH size and the presence of midline shift. These outcome variables were determined by a priori literature demonstrating their importance in determining CSHD patient eligibility for acceptance for neurosurgical intervention.

### Statistical analysis

All statistical analysis was performed using the IBM SPSS software (Statistical Package for the Social Science; SPSS Inc., Chicago, IL, USA) Version 28 for Mac and the Python programming language (Version 3.10). Independent samples t-tests were used to compare all continuous variables and Chi squared tests were conducted for all categorical variables. Relative risk (RR) and effect size analysis using odd’s ratio (OR) was conducted to compare the risk of developing the aforementioned outcomes in the DOAC and warfarin groups. A *p* value < 0.05 was considered statistically significant.

## Results

### Baseline patient characteristics

The mean age of the patients was 78.5 ± 11.5 years, with a male predominance (67.3%)**.** In this cohort, 905 patients (33.8%) were on antithrombotic therapy, with 298 patients (11.1%) on warfarin and 203 patients (7.6%) on DOACs. Cardiac rhythm disorder was the most common cause for antithrombotic therapy, followed by ischaemic heart disease, cardiac valvular disease and peripheral vascular disease (Table 1). Majority of patients had a smaller sized haematoma (41.2%), with 30.3% patients presenting with moderate sized CSDHs and 28.6% had large sized haematomas. Midline shift of the brain parenchyma was present in 775 patients. A third of the total cohort of patients (*n* = 891) had a reduced consciousness level (GCS < 15), with 181 patients (6.8%) with a GCS < 13. Off the total patients presenting with a CSDH, 750 patients (28%) were accepted by the neurosurgical team for intervention, in which 219 of these patients were on anticoagulation: 82 patients on warfarin and 38 patients on DOACs.

**Table 1:** CSDH cohort demographic, reason for antithrombotic therapy, and characteristics of the chronic subdural haematoma. Categorical Variables Represented as n (%), Continuous Variables Represented as Mean (± Standard Deviation)

Demographics summary table	
Total number of patients	2675
Average age, years ± SD	78.5 ± 11.5
Male patients (%)	1799 (67.3)
Female patients (%)	876 (32.7)
Number of patients on antithrombotic therapy	905 (33.8)
**Reason for antithrombotic therapy**	**Number of patients with condition (%)**
Ischaemic Heart Disease	362 (13.5)
Cardiac rhythm disorder	545 (20.4)
Cardiac valvular disease	110 (4.11)
Peripheral vascular disease	97 (3.6)
**Type of antithrombotic medication**	**Number of patients on antithrombotic medication (%)**
Warfarin	298 (11.1)
DOAC	203 (7.6)
Aspirin	202 (7.55)
Antiplatelet	148 (5.5)
LMWH	54 (2.02)
**CSDH Characteristics**	**Number of patients with condition (%)**
Glasgow Coma Score (GCS)	
GCS 15 (%)	1784 (66.7)
GCS 14 (%)	545 (20.4)
GCS 13 (%)	165 (6.2)
GCS <13 (%)	181 (6.8)
CSDH Size	
Small (%)	1101 (41.2)
Moderate (%)	810 (30.3)
Large (%)	764 (28.6)
Midline shift (%)	775 (28.97)

### Outcome analysis

**Fig. 1:**
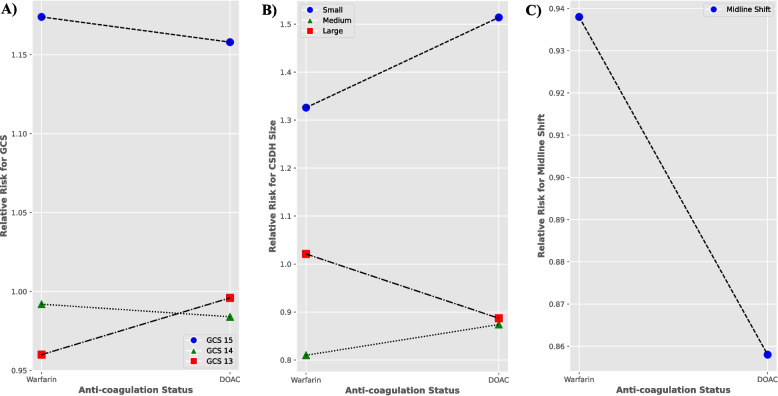
Comparative relative risk analysis between DOAC and Warfarin for various CSDH clinical and radiological outcomes: **A**) GCS, **B**) CSDH size and **C**) Midline Shift. GCS = Glasgow Coma Score

Female sex and antithrombotic therapy were significantly associated with CSDH size (*p* = 0.0004, *p* < 0.0001), midline shift (*p* < 0.0001, *p* < 0.0001) and GCS (*p* = 0.0367, *p* = 0.0073). There was a statistically highly significant association between the type of antithrombotic therapy and both midline shift and size of the CSDH (*p* < 0.0001), but not GCS levels (*p* = 0.1956) (Table 2). RR analysis demonstrated a lower risk of developing a larger sized hematoma (0.887 vs 1.021) and a reduced likelihood of midline shift (0.858 vs 0.938) if a patient was anticoagulated using DOACs as opposed to warfarin (Table 3), and this was further supported by effect size analysis using OR analysis (Table 4). OR analysis revealed that there was reduced odds of developing a larger sized haematoma (0.636 (*p* = 0.009, 95% confidence intervals (CI) 0.451–0.895) vs 1.069 (*p* = 0.615, 95% CI = 0.825–1.386)) and midline shift (0.524 (*p* = 0.0005, 95% CI 0.363–0.756) when on DOACs compared to warfarin. No significant difference in RR for impaired GCS was found between DOACs or warfarin (Fig. 1.A). A small increase in the likelihood of developing a small/moderate sized haematoma was observed on DOACs compared to warfarin (1.514 vs 1.326, 0.874 vs 0.810) as shown by Fig. 1.B. This was observed in the OR analysis as well (2.297 (*p* = < 0.0001, CI 1.715–3.077) vs 1.824 (*p* = < 0.0001, CI 1.432–2.324), 0.516 (*p* = 0.0005, CI 0.357–0.748) vs 0.376 (*p* = < 0.0001, CI 0.268–0.527)). Comparative risk analysis between DOACs and warfarin as per Fig. 1.C, further demonstrated a higher risk of midline shift for patients on warfarin (RR 1.431, 95% CI 0.998–2.05, Cohen’s d 1.95), that trended towards statistical significance (*p* = 0.0511). Warfarin use was seen to result in a statistically significant (*p* = 0.0259) increased likelihood of neurosurgical acceptance for surgical intervention (RR 1.473, 95% confidence interval 1.05–2.07) compared to DOACs.

**Table 2:**
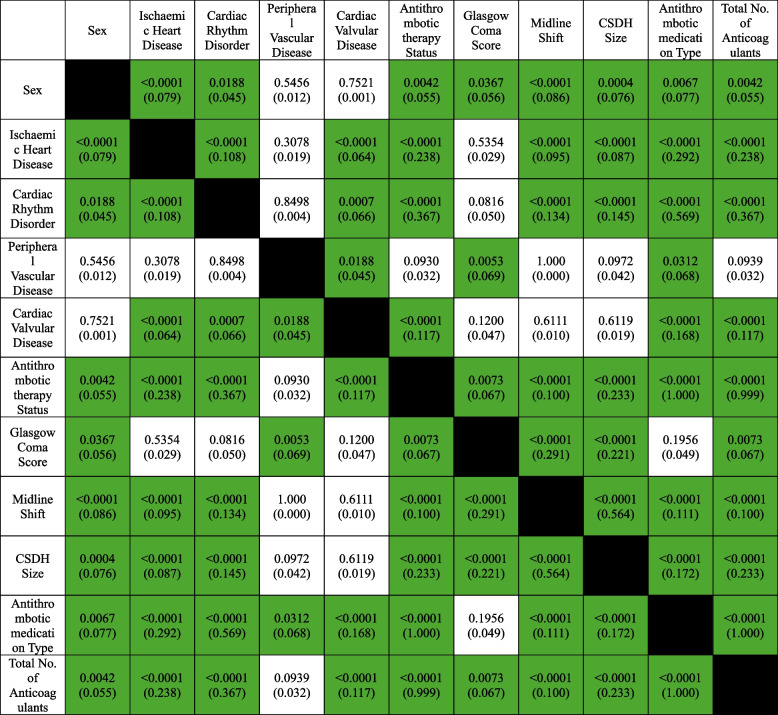
Results of chi-square and independent samples t-tests assessing association between predictive variables and CSDH characteristics. *P*-values are provided, with the Cramer V in parenthesis

**Table 3:** Relative Risk, 95% confidence intervals and *P*-values for Warfarin and DOAC & CSDH characteristics of interest

Type of anticoagulation		GCS				CSDH Size			Midline Shift
		15	14	13	< 13	Small	Moderate	Large	
**Warfarin**	**RR**	**1.174**	**0.992**	**0.994**	**0.960**	**1.326**	**0.810**	**1.021**	**0.938**
	95% CI Intervals	0.901–1.529	0.733–1.341	0.593–1.668	0.502–1.838	1.040–1.689	0.578–1.136	0.787–1.323	0.711–1.238
**DOAC**	**RR**	**1.158**	**0.984**	**0.970**	**0.996**	**1.514**	**0.858**	**0.887**	**0.858**
	95% CI Intervals	0.846–1.586	0.685–1.415	0.448–2.096	0.556–1.784	1.130–2.027	0.592–1.242	0.630–1.249	0.595–1.238

**Table 4:** Odd’s ratios, 95% confidence intervals and *P*-values for Warfarin and DOAC & CSDH characteristics of interest

Type of anticoagulation		GCS				CSDH Size			Midline Shift
		15	14	13	< 13	Small	Moderate	Large	
**Warfarin**	**OR**	**1.262**	**0.961**	**0.911**	**0.448**	**1.824**	**0.376**	**1.069**	**0.790**
	95% CI Intervals	0.969–1.645	0.710–1.299	0.543–1.528	0.234–0.858	1.432–2.324	0.268–0.527	0.825–1.386	0.599–1.042
	*P*-value	0.084	0.794	0.724	**0.015**	**<0.0001**	**<0.0001**	0.615	0.095
**DOAC**	**OR**	**1.238**	**0.924**	**0.523**	**0.938**	**2.297**	**0.516**	**0.636**	**0.524**
	95% CI Intervals	0.904–1.696	0.643–1.328	0.242–1.131	0.524–1.681	1.715–3.077	0.357–0.748	0.451–0.895	0.363–0.756
	*P*-value	0.183	0.669	0.099	0.831	**<0.0001**	**0.0005**	**0.009**	**0.0005**

## Discussion

To our knowledge, this is the first study analysing the impact of oral anticoagulation, comparing warfarin and DOACs, specifically on the characteristics of CSDH, such as the size of the haematoma, presence of midline shift and GCS level at presentation. These clinical and radiological features have been previously demonstrated to be important predictors of patient acceptance for neurosurgical intervention [8, 9]. We observed highly significant associations between anticoagulant type and CSDH features, with RR analysis revealing a reduced likelihood of developing a larger sized hematoma and midline shift with the use of DOACs compared to warfarin. The RR analysis for GCS levels showed minimal differences between DOACs and Warfarin. Effect size analysis further supported this difference with DOACs demonstrating a reduced odds of resulting in a larger haematoma and midline shift. Warfarin was more likely to cause a larger midline shift on comparative risk analysis, though this only approached statistical significance.

As per prior literature, our CSDH patient cohort was elderly (mean age of 78.5 years) and majority male; both known risk factors of the development of CSDH [4]. Similar to literature, a 2:1 male to female CSDH bias was observed [1, 10, 11]. The primary reason for increase in CSDH risk with age is thought to be due to increase in brain parenchymal atrophy with age and subsequent stretching of bridging veins, that are vulnerable to damage, rupture and haemorrhaging into the sub-dural space [1, 4, 11–13]. However, the male CSDH preponderance seen in literature, which has historically been attributed to increased likelihood of head trauma and chronic alcoholism contributing to increased brain atrophy [1, 10, 12] has been questioned [11]. Interestingly, female sex was associated with ischaemic heart disease, cardiac rhythm disorder, antithrombotic therapy and the CSDH characteristics of interest in our study. Similarly, Marshman et al. [11] found that risk factors for CSDH formation generally trended towards females [11], and Gaist et al. [3] observed a higher relative risk of subdural haematomas in women than men associated with antithrombotic drugs (aspirin and vitamin K antagonist (VKA)) in Denmark [3]. The underlying mechanism behind the male CSDH bias, despite a paradoxical bias of CSDH risk factors towards females, remains to be fully explained [11, 14].

Whilst head trauma had been previously considered the most important and common risk factor [15, 16], the widespread use of anticoagulant and antiplatelet medication has resulted in an increase in CSDH incidence, at times secondary to only minimal cranial trauma [4, 5, 12]. A study evaluating the prescription trends in oral anticoagulants across England between 2009 – 2019 showed that DOACs accounted for 61.8% of all oral anticoagulants prescribed in 2019, compared to 16.4% in 2015. Geographical differences in anticoagulant prescriptions between different clinical commissioning groups were also reported. Per 1000 population, Greater Manchester (GM) had approximately 130 prescriptions of warfarin and ~ 200 prescriptions of DOACs in 2019. Thus, it can be inferred that approximately 372,809 individuals were on warfarin and 573, 553 individuals were on DOACs: ~ 33% of the total GM population on oral anticoagulation in 2021 [17]. Considering the latest NHS England initiative to increase DOAC prescription for stroke prevention [18], these numbers are likely on the conservative side. Inferring from incidence data (48/100,000 [2]), ~ 1376 patients would have presented with CSDH in the 2021 population of GM. Based on our data, ~ 465 (33.8%) of these patients would have been on oral anticoagulation. It is in our experience that nearly all cases of detected CSDH in the community are referred to the neurosurgical unit, with our current data suggesting that 28% of these cases would be accepted for intervention.

Importantly, the type of antithrombotic therapy used was found to be a predictor of CSDH characteristics in this study, with a significant association with the size of the CSDH and the presence of midline shift. However, GCS was not found to be statistically associated with the type of antithrombotic drug. This could have been because of the variability in clinical presentation frequently observed in CSDH patients, ranging from asymptomatic to severely reduced GCS scores, wherein the majority of patients present with a GCS between 13–15 [15, 19, 20]. Likewise, 93.2% of our patient cohort presented with a GCS score > 13. Additionally, we did not observe any difference in relative risk between different types of antithrombotic medication and a GCS score of < 13. Notably, no statistical difference in GCS scores were found for CSDH patients transferred for neurosurgical intervention versus those who were not in a multicentre, prospective study in the UK [20].

Investigating the association observed between the type of antithrombotic therapy and both the size of the haematoma and presence of midline shift using RR analysis revealed that being anticoagulated with warfarin resulted in a higher risk of forming a larger haematoma (RR 1.021) when compared to DOACs (RR 0.887). It has been established that warfarin use is highly associated with an increased risk of CSDH [3, 5, 21, 22], with a meta-analysis odds ratio (OR) of 2.9 when compared to direct factor Xa inhibitors [21]. Studies into the effect of warfarin on intracerebral haemorrhages (ICHs) has also demonstrated that the use of warfarin results in larger sized haematomas [23, 24]. In both spontaneous and anticoagulant-associated ICHs, the size of the haematoma is considered the most powerful predictor of neurological deterioration, functional outcomes and 30-day mortality [25–27]. Warfarin-associated ICHs were also seen to be more likely to expand, and haematoma expansion is linked with increased mortality and negative functional outcomes [24, 28, 29]. An international normalised ratio (INR) > 3 is thought to be associated with a larger ICH volume and increased mortality rates [23, 30, 31]. Interestingly, antithrombotic therapy (including warfarin and DOACs) resumption does not seem to be associated with an increase in the risk of CSDH recurrence [32–34].

Gaist et al. [3] found that the use of a VKA resulted in a higher likelihood of fatal subdural haematomas when compared to DOACs (adjusted OR of 6.30 vs 3.87) in their case–control study of 10,010 patients. The concurrent use of VKAs and antiplatelet therapy was seen to increase the risk of subdural haematomas incidence by ~ eightfold. In their regional sub-group analyses of 517 subacute or chronic subdural haematoma cases, VKAs were shown to result in a higher risk of developing acute, sub-acute and chronic subdural haematomas, especially in low to moderate trauma severity, when compared to low-dose aspirin. VKA users had a higher 30-day mortality after subacute or chronic subdural haematomas compared with never users of anticoagulation. However, it must be noted that they did not differentiate between subacute and chronic subdural haematomas and classed it together in their sub-group analyses, and also excluded non-VKAs. Thus, any CSDH-related conclusions drawn from this study should be interpreted with caution. Our study is the first and largest to show that warfarin usage is likely to result in a larger volume haematoma in the specific context of CSDHs, when compared to DOACs. This is further reflected in our comparative risk analysis finding that the use of warfarin trended towards a higher likelihood of midline shift as well, likely as a result of the mass effect exerted by the larger haematoma volume. This is in agreement with Gaist et al. (2017) that found a higher likelihood of midline shift on VKAs (OR 3.29) compared to never users of anticoagulation [3]. The presence of a large sized haematoma and midline shift represent an increased likelihood of impending brain compression and damage that require urgent neurosurgical input and interventions [8]. This is further supported by our comparative risk analysis that showed that patients on warfarin were more likely to be accepted for intervention under the neurosurgical team when compared to DOACs, as they are more likely to present with these high risk CSDH features.

Interestingly, when on DOACs, a small increase in the likelihood of developing a small/moderate sized CSDH was observed when compared to warfarin in our study. DOAC-associated ICHs have been found to have smaller haemorrhage volumes, less haematoma expansion and lower mortality rates when compared to warfarin [27, 35, 36]. The underlying mechanism leading to these findings requires clarification. Upon head trauma, often minimal in CSDH patients, vascular injury may occur, and tissue factor (TF), which is present in high levels around blood vessels in the brain [37], is expressed by endothelial cells and binds with factor VIIa, forming TF-VIIa complexes. This activates factor IX and factor X, finally resulting in fibrin formation. However, warfarin inhibits the hepatic synthesis of vitamin K-dependent coagulation factors II, VII, IX and X and regulatory factor protein C and S, disrupting the extrinsic coagulation pathway [38]. The reduced levels of factor VII available to bind with TF impairs the normal haemostatic mechanism. Whereas DOACs act by competitively inhibiting only factor Xa or thrombin and has no effect on TF-VII complex formation [39]. Additionally, warfarin has a half-life of 36 – 42 h [38], whereas DOACs are rapidly absorbed and have a plasma elimination half-life of ~ 12 h [39, 40]. We hypothesise that following insult to small, bleeding-prone cerebral vessels, DOACs-associated CSDHs may initially haemorrhage more into the subdural space, but does not expand, and remains small/moderate in volume, whilst warfarin may lead to a slow accumulation and a larger haematoma over time which can expand and exert mass effect, causing midline shift of brain parenchyma, ultimately resulting in unfavourable neurological, functional and mortality outcomes for the patient.

### Limitations

Despite these results, our study still has some limitations. Firstly, this study is focussed on CSDHs referred to neurosurgery rather than CSDHs prevalent in the community. However, it is our experience in the UK health service that the vast majority of CSDHs detected in the community are indeed referred to neurosurgery. Additionally, despite the multi-centre nature of data included in the study, it would also be valuable to confirm our findings in larger cohorts and randomised controlled trials. Secondly, a more inclusive set of predictor variables could have been analysed. Pre-operative co-morbidities that could have contributed to an increased likelihood of brain atrophy such as stroke, dementia, hypertension, coagulopathies, trauma severity and alcohol intake can be important to consider potential confounding factors. Thirdly, whilst we compared the most commonly used oral anticoagulation medications, the effects of other antithrombotic therapy, namely antiplatelet, low-molecular weight heparin and aspirin, were not explored in detail. The correlation between INR with the initial CSDH volume and presence of midline shift was also not assessed. Similarly, renal function tests and DOAC plasma levels were not routinely collected in the neurosurgical referral database, and as such, the effect of these variables was not assessed in this current paper. Therefore, it would be useful to perform a multivariate regression analysis in future studies incorporating these confounding variables. We did not analyse the differences in symptoms in CSDH patients between those on warfarin and those on DOACs. It is also not the case that those being treated conservatively were asymptomatic as there are other confounding variable such as co-morbidities, frailty and advanced care plans that would have influenced decision making. Lastly, whilst we focussed on variables shown to be important in influencing neurosurgical acceptance for surgical intervention, an exhaustive clinical outcome set was not utilised. Thorough details regarding the management for patients after their CSDH was discovered falls beyond the scope of the current paper. It would be valuable in the future to investigate the medical ± surgical management of these patients, rates of CSDH recurrence and mortality rates, but also neurological functional outcomes using the Glasgow outcome scale-extended or modified Rankin scales [41, 42], and radiological outcomes through post-operative scans, although difficult in a resource limited public healthcare system. Performing comparative analysis of surgical outcomes is vital to future studies on the topic.

## Conclusion

In conclusion, this is the first and largest ever study comparing the clinical and radiological characteristics of CSDHs important for neurosurgical intervention as a result of the use of DOACs and warfarin. Along with their well evidenced safety profile, DOACs have the potential to be a safer method of anticoagulation in the elderly population when compared to warfarin in the context of CSDHs, as although there was no significant difference in GCS between the groups, they appear to be linked to the development of smaller sized hematomas and reduced midline shift. However, further research on mortality and functional outcomes is needed to draw a definitive conclusion. This is particularly relevant for an ageing population with multi-comorbidities on increasing amounts of anticoagulation medication. These findings have the potential to contribute to the development of future anticoagulation guidelines for such patients, influence disease management and ultimately enhance patient care.

## Data Availability

The datasets generated and/or analysed during the current study are not publicly available due local information governance policies but are available from the corresponding author on reasonable request.

## Abbreviations

CSDH: Chronic Subdural Hematoma
DOAC: Direct Oral Anti-Coagulant
GCS: Glasgow Coma Scale
GM: Greater Manchester
ICH: Intracerebral Haemorrhage
OR: Odd’s Ratio
RR: Relative Risk
TF: Tissue Factor
UK: United Kingdom
VKA: Vitamin K antagonist
